# Health status and health behaviour of the Hungarian homeless people

**DOI:** 10.1186/s13690-021-00534-2

**Published:** 2021-02-02

**Authors:** Emese Nagy-Borsy, Zsolt Vági, Petra Skerlecz, Blanka Szeitl, István Kiss, Zsuzsa Rákosy

**Affiliations:** 1 Department of Public Health Medicine, Medical School, University of Pécs, Szigeti út 12, Pécs, 7624 Hungary; 2 TÁRKI Social Research Institute, Budapest, Hungary

**Keywords:** Homeless, Health status, Health behaviour, Hungary, ETHOS classification

## Abstract

**Background:**

Homelessness has risen recently in Europe, but there is lack of comprehensive health data on this population. Our aim was to characterize the health of the Hungarian homeless population.

**Methods:**

We performed a health survey with 453 homeless individuals. The results were compared to the age and sex standardized data of the general Hungarian population and its lowest income quintile from the European Health Interview Survey 2014. The differences by the ETHOS classification within the homeless population were also studied.

**Results:**

Significantly fewer homeless people reported good health status than in the general population or in its lowest income quintile (*p*< 0.001). Of the participants 70% had at least one chronic disease, only 41% of them visited a GP and 35% took medication in the previous 12 months. While 59% of the lowest income quintile and 50% of the general population had at least one chronic disease, almost all of them visited a physician and took medication. The highest prevalence of morbidity (80%) and multimorbidity (46%) was reported in the houseless group. The majority of the homeless people were current smokers, the prevalence was much higher than in the two reference populations (*p*< 0.001). The prevalence of heavy drinkers was the highest among the roofless participants (40%).

**Conclusions:**

Homeless people have much poorer health and they utilize health services less than the most disadvantaged quintile of the general population. There is a clear social gradient within the homeless population, as well, which calls for integrated approaches for specific interventions to improve their health.

**Supplementary Information:**

The online version contains supplementary material available at 10.1186/s13690-021-00534-2.

## Background

Homelessness is a complex phenomenon. It can be triggered by different individual (e.g. poverty, mental health problems including substance misuse, violence) and structural factors (e.g. losing jobs, absence of low-cost housing, lack of employment opportunities for low-skilled workers and income support) [1]. The number of homeless people is increasing in the European Union (EU). Approximately 400,000 people are homeless on any given day [2]. More and more people are appearing in the homeless shelter system in Hungary too, according to the data of the annual ‘Third of February Homeless Survey’. In 2020, it is reported that 7604 people used homeless shelters on the day of the survey [3]*.*

Homelessness is not only a social or political issue but a public health concern, too. It is well known that financial deprivation has a strong association with poor health status. People in this vulnerable and isolated situation are particularly affected by health problems which are associated with higher rates of premature mortality [4–7]*.* Harsh living conditions, including the street and crowded shelters, increase the risk of infectious diseases like hepatitis C and HIV [8, 9], respiratory infections, hepatitis B [10] or different skin infections (pediculosis, scabies) [11]. As a consequence of the ageing of the population, the number of elderly people who experience homelessness is also increasing in many developed countries, which has implications for the prevalence of age-related chronic diseases in the homeless population [2]*.* Homeless people are also at greater risk of developing multiple morbidities [12], including respiratory and circulatory conditions [13–15]; injury (particularly through violence) [16], poor oral health [17], feet problems [2]. Homeless individuals have difficulties managing diseases, in some countries many of them do not have health insurance, they cannot access specific medical care, and they are more likely to use the emergency services [18, 19]. Moreover, the material deprivation and the lack of resources limit their capacity to focus on their health problems and health care needs, and it prevents them from the health promoting behaviour, as well. The prevalence of smoking, alcohol, and drug addiction is higher in the homeless population than in the general population [20–24]. Material deprivation also limits their ability to purchase and take medications and to manage health issues that otherwise are relatively easy to control [25].

The complexity of homelessness, the different homeless care systems in the EU Member States, and the lack of harmonized health related data of homeless people at EU level is a major challenge in identifying and understanding the specific health problems of this vulnerable group. Although many studies have documented poor health among homeless people, few studies have compared their health with that of the general population. This lack of comparison is an especially important issue in Central Eastern Europe, where the health status of the population is worse, characterized by high premature mortality. So far comparative analysis with the average Hungarian population data is available for the homeless population only for Budapest from 2002 [26].

The effect of the social gradient is well known on life expectancy, health status and health behaviour [27]. In public health, homeless people are usually considered as a homogeneous disadvantaged group. Usually, the health problems of roofless people are generalized to the whole homeless population. However, a social gradient exists within this group as well, according to their living conditions and time spent as homeless.

Our aim was to address the specific health challenges and health related behaviour of people who used the Hungarian homeless shelter system, and to compare their characteristics according to the ETHOS categories, and additionally to the data of the general Hungarian population and its lowest income quintile.

## Methods

### Homeless health survey 2015

Only homeless people who used homeless services were considered as the target population. A convenience sample of homeless people was involved in the study from major Hungarian cities. Various charity organizations were involved in the study. Homeless participants were interviewed by the researchers in Pécs in the day centres, in the night shelters and in the medical institution of the homeless service system of TÁMASZ Foundation; in Debrecen in the day centre, in the night shelters and in the temporary accommodation of the ReFoMix homelessness service, in the day centre of the Reformed Charity Service, in the day centre of the Hungarian Interchurch Aid; in Budapest in the day centres, in the night shelters, in the temporary accommodation and in the disinfectant bath institute of the Hungarian Maltese Charity Service. The selection of these institutions was performed in an ad hoc way, as these institutions were willing to cooperate in the research. All homeless people who were cared for in these institutions at the time of the study were interviewed. None of them refused to participate. The European Observatory on Homelessness and the European Federation of National Organisations Working with the Homeless (FEANTSA) have developed a conceptual classification called European Typology on Homelessness and Housing Exclusion (ETHOS). We used this typology to classify the studied population based on their living situations. The typology includes four distinct housing situations covering all forms of living situations of homelessness across Europe: “rooflessness (without a shelter of any kind, sleeping rough), houselessness (with a place to sleep but temporary in institutions or shelter), living in insecure housing (threatened with severe exclusion due to insecure tenancies, eviction, domestic violence) living in inadequate housing (in caravans on illegal campsites, in unfit housing, in extreme overcrowding)” [28]. Although the ETHOS has been acknowledged as the standard definition of homelessness on the Jury of the European Consensus Conference on Homelessness 2011, it is not fully adopted by every EU country. In Hungary, according to the law, homeless people are persons without any registered place of residence or those whose registered place of residence is the accommodation for homeless people.

The data was collected from the homeless respondents using an anonymous questionnaire which was administered by the interviewers.

Respondents were involved in the research voluntarily. This study complies with the criteria of the Scientific and Research Ethics Committee of the Medical Research Council, Hungary (registration number: 4648//2015/EKU). All participants gave written informed consent.

### National Health Interview Survey 2014 [29]

We compared the health data of homeless people to the health data of the Hungarian general population and of the lowest income quintile obtained from the Hungarian part of the European Health Interview Survey 2014 (EHIS2014).. In the analysis, the data of this survey were age- and sex-standardized for the homeless population to allow an unbiased comparison of the two populations.

### Questionnaire

Most of the questions of the two surveys were the same or directly comparable. The questionnaires covered socioeconomic status, health behaviour, self-reported health status, chronic diseases, health service utilization, and medication use.

Sociodemographic factors included age, gender, education level, economic activity, marital status, and characteristics of homelessness. We grouped the education level into five categories: primary school not finished, primary school, vocational school, high-school graduation, and college/university degree. Economic activity was categorized as unemployed, have a job or retired. Marital status was categorized as married or civil partnership, unmarried, widow (er) and divorced.

Questions on health behaviour included alcohol consumption and smoking habit. Body height was measured standing upright to the nearest 0.1 cm (cm) using a 2 m (m) wall-mounted stadiometer roll-up height measurer. Bodyweight was self-reported in the reference population and measured using OMRON body compositor monitor BF 511 in the homeless population. Body mass index (BMI) was calculated as body weight (kg) divided by height in meters squared (m^2^). BMI was categorized as abnormally thin (BMI< 18.5 kg/m^2^), normal (BMI=18.5–24.99 kg/m^2^), overweight (BMI=25–29.99 kg/m^2^), or obese (BMI ≥30 kg/m^2^), in accordance with the WHO guidelines. The frequency of alcohol consumption was asked by the question “During the past 12 months, how often have you consumed any kind of alcoholic drinks?” The four categories of alcohol consumption were defined as abstinent, occasional drinker, moderate drinker, and heavy drinker. People were categorized as abstinent who did not report alcohol consumption at all. Those belonged to the occasional drinker category, who reported less than weekly alcohol consumption. Heavy drinker was defined as alcohol consumption several times a week. Persons consuming alcohol but not qualifying as heavy or occasional drinkers were classified as moderate drinkers. The quantity and the type of consumed alcohol were asked by the question “How many and what kind of alcoholic drinks do you have each day in a typical week when you drink?” Smoking habit was categorized as “never smoker”, “former smoker” and “current smoker”. The amount of cigarettes was assessed by the question “On average, how many cigarettes do you smoke each day?”

The self-reported health status was assessed by the question “How is your health in general?” The answer categories were: very good, good, fair, poor, very poor, and do not know, but in our analysis very good and good, and very bad and bad were combined. Because of the low health literacy of homeless people, the presence of chronic diseases was asked differently in the homeless survey than in the general health survey. First, we asked the question “Do you have any longstanding disease or health problem?” without specifying the different chronic diseases. The data corresponding to this question could be compared to the general population, as a similar question was asked in the national health survey. Next, the presence of the chronic diseases was asked from the participants and it was also assessed in the health records obtained from the general practitioners (GP) caring for homeless people in the participating institutions. This information was lacking for the general population. The diseases were categorized according to the International Classification of Diseases. The frequency of GP visit was assessed by the question “When was the last time you consulted a GP on your own behalf?”

Regarding to the dental status, the following questions were asked: “How is your dental status in general?”, “How many missing teeth do you have?” and “Do you have any prosthesis?”

### Sampling and statistical analysis

The target populations of the two surveys were complementary to each other: the EHIS2014 survey included only individuals living in private households, thus only homeless individuals living in insecure housing were part of the sampling frame in theory. However, many of these individuals are not officially registered at the address where they live thus in practice they did not belong to the sampling frame of the national health survey. On the other hand, the homeless population survey included individuals who utilized certain types of homeless care services.

To control for confounding by age and sex, the estimates from the national survey were weighted by the age and sex distribution of the homeless population*.* The data of the two surveys were then integrated and the analysis was performed on this combined and weighted database. Income was not included into the variables used for weighting because the exact amount of income was not available for EHIS2014 data (only quintiles), so weighting was not possible. Therefore, besides comparing the characteristics of the homeless population to the weighted total of the general population, we also compared them with the lowest income quintile of the weighted total population.

Chi-square test was used for hypothesis testing with a significance level of 5%.

The maximum margin of error (half of the width of the 95% confidence interval) of the prevalence estimates was determined by the following formula [30]: $\frac{0.98}{\sqrt{n.}}$

Where *n* is the sample size. In our case, the maximum margin of error for estimates for the homeless population was ± 4,6%, for the general population ± 1,3%, for estimates of the lower income quintile of the general population ± 2,5%.

## Results

In total, 453 homeless adults, roughly 1% of the entire Hungarian homeless population, were interviewed between 1 January and 30 April in 2015. The sample size of the National Health Interview Survey was 5826. The survey was conducted between September 15–December 15, 2014. The proportion of males was much higher in the homeless study population (81%) than in the representative Hungarian sample (46%). The age distribution of the homeless sample was shifted towards the older age groups (52 years, range: 18–89 years). The distribution of each of the examined characteristics differed statistically significantly in the homeless population from the distribution in the general population and in its lower income quintile.

Fifty-two percent of the participants reported being homeless for more than 5 years, 32% lived as homeless for 1–5 years, and 13% became homeless within a year. The majority of them (61%) was homeless for first time, 21% could have one or two occasions not in homeless status for shorter or longer period, 16% had experienced multiple episodes of homelessness, 2% did not answer this question. According to the European typology of homelessness, 27% of respondents were roofless people, 51% were houseless, 11% belonged to the insecure housing group, 11% lived in inadequate housing conditions. The majority (64%) of roofless individuals lived as homeless for more than five years. The proportion of individuals being homeless for less than one year was highest (17%) in the inadequate housing category.

### Socioeconomic status

Socioeconomic characteristics are summarized in Table 1. The level of education was considerably lower among homeless people than the national references (Table 1). High proportion of homeless individuals (51%) had only primary education compared to the 31% of the lowest income quintile of the general population and 13% of the general population.

**Table 1:** Socioeconomic status in the homeless population and in the general population in Hungary

	Homeless population						Lowest income quintile of the general population		General population	
	Roofless	Houseless	Insecure housing	Inadequate housing	*P*-value*	Overall		P-value†		P-value‡
**Education degree (%)**										
Primary school is not finished	3.2	8.8	10.4	4.1	0.07	6.9	4.8	< 0.001	1.9	< 0.001
Primary school	51.6	52.2	45.8	53.1		51.4	30.6		13.3	
Vocational school	33.9	28.5	37.5	30.6		31.2	50.5		47.9	
High-school graduation	7.3	7.0	6.3	0		6.2	11.0		18.0	
College/University degree	4.0	3.5	0	12.2		4.2	3.1		18.9	
**Economic activity (%)**										
Unemployed	81.5	80.6	77.1	77.6	0.9	80.1	36.9	< 0.001	17.5	< 0.001
Have a job	9.7	10.8	14.6	16.3		11.5	40.7		61.8	
Retired	8.9	8.6	8.3	6.1		8.4	22.4		20.7	
**Marital status (%)**										
Married or civil partnership	12.9	8.4	16.7	18.4	0.02	11.6	77.5	< 0.001	62.7	< 0.001
Unmarried	43.5	34.1	45.8	46.9		39.4	15.2		20.5	
Widow/widower	8.1	8.4	4.2	0		6.9	2.7		5.3	
Divorced	35.5	49.1	33.3	34.7		42.1	4.7		11.5	

*Comparing the different homeless categories

† Comparing the overall homeless category to the lowest income quintile of the general population

‡ Comparing the overall homeless category to the general population

The proportion of active workers and the family status markedly differed in the two populations. Unemployment was much higher in the homeless population (80%) than in lowest income quintile of the general population (37%) and in the general population (18%). The prevalence of unemployment was the highest among the roofless people. The percentage of retired persons was 8% of the surveyed homeless persons. This proportion was similar and much larger in the two reference populations, 21 and 22%, respectively.

Almost half (42%) of the homeless people were divorced, this rate was almost ten times higher than in the lowest income quintile of the general population (5%) and almost four times higher than in the general population (12%). The proportions of widows/widowers and unmarried people were also higher in the homeless population. People living in partnership was the lowest in the houseless group.

### Self-reported health status, prevalence of chronic diseases and usage of health care

The self-reported health status of the homeless people was much worse than of the general population (Table 2). Significantly fewer homeless people reported their health status as good or very good and many more reported bad or very bad health status than in the general population or in the lowest income quintile of the general population. In addition, people who lived on the street or in shelters considered their health poorer than who lived in inadequate or insecure housing condition. There was no significant association between the self-reported health status and the length of homelessness. Of the participants, 79% reported their dental status as poor, this rate was much higher than in the general population (26%) and in the lowest income quintile of the general population (42%). Almost all homeless people (96%) had missing teeth but only 8% of them had a prosthesis, most of them lived in inadequate housing conditions (Table 2).

**Table 2:** Prevalence (%) of self-reported health status, chronic diseases, medication, general practitioner (GP) visit and oral health status in the homeless population and in the general population in Hungary

	Homeless population						Lowest income quintile of the general population		General population	
	Roofless	Houseless	Insecure housing	Inadequate housing	P-value*	Overall		P-value†		P-value‡
**Self-reported health status**										
Good/very good	2.4	4.3	6.3	14.3	0.001	5.1	36.4	< 0.001	56.4	< 0.001
Fair	56.5	46.3	60.4	51		51.1	36.7		30.9	
Bad/very bad	37.9	35.1	16.7	24.5		32.7	26.9		12.7	
Do not know	3.2	14.3	16.7	10.2		11.1	0		0	
**Self-reported oral health status**										
Poor dental status	79.7	79.6	72.9	81.6	0.7	79.1	42.0	< 0.001	25.6	< 0.001
Having missing tooth	96.7	96.9	88.9	97.8	0.12	96.1	81.0	< 0.001	70.9	< 0.001
Having a prosthesis	5.7	8.5	8.5	10.9	0.7	8.0	38.1	< 0.001	48.5	< 0.001
**Prevalence of self-reported chronic diseases**	76.6	85.3	56.3	73.5	< 0.001	70.4	59.2	< 0.001	49.5	< 0.001
**Prevalence of comorbidity**	36.3	46.1	41.7	20.4	0.007	40.1	23.5	< 0.001	14.3	< 0.001
**Medication**	25.0	37.2	25.0	22.5	< 0.001	30.9	56.8	< 0.001	63.3	< 0.001
**Medication among people with chronic disease**	37.2	45.7	39.3	35.7	0.3	34.8	92.4	< 0.001	91.8	< 0.001
**GP visit**										
Within 12 months	33.1	42.4	35.4	28.6	0.22	37.6	77.7	< 0.001	75.6	< 0.001
12 months or earlier	57.3	48.9	52.1	53.1		52.0	21.3		23.8	
Never	9.7	8.7	12.5	18.4		10.4	1.0		0.6	
**GP visit among people with chronic disease**										
Within 12 months	43.6	48.4	39.3	46.4	0.9	41.4	92.8	< 0.001	91.4	< 0.001
12 months or earlier	47.4	44.6	53.6	46.4		51.3	7.2		8.4	
Never	9.0	7.1	7.1	7.1		7.3	0		0.2	

*Comparing the different homeless categories

† Comparing the overall homeless category to the lowest income quintile of the general population

‡ Comparing the overall homeless category to the general population

**Fig. 1:**
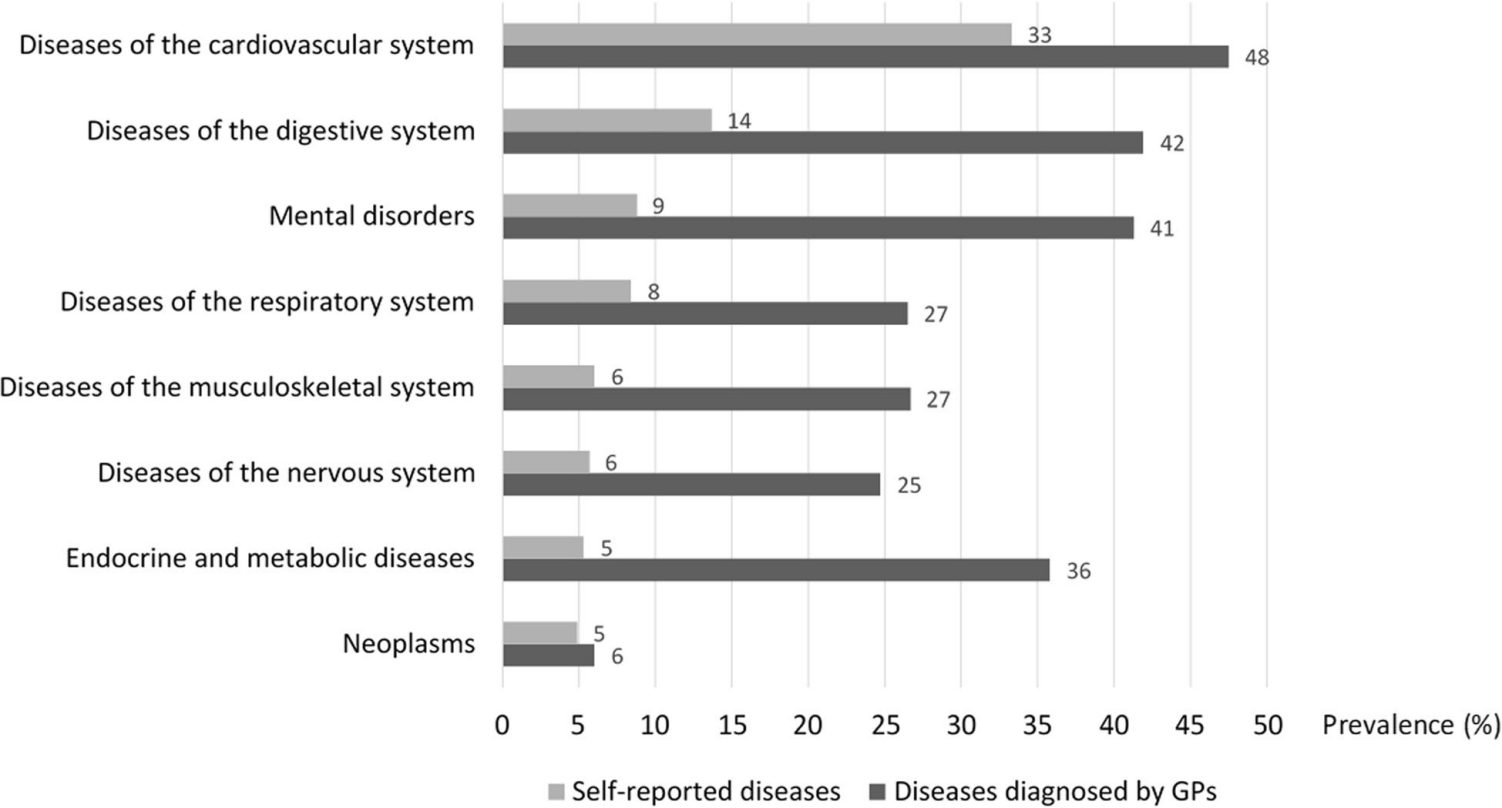
Self-reported diseases and diseases diagnosed by general practitioners in the homeless population

Of the general population 50%, of the lowest income quintile 59%, and of the homeless individuals 70% reported to have at least one chronic disease. Among the homeless participants 33% had cardiovascular, 14% digestive system, 9% mental, 8% respiratory, 6% musculoskeletal, 6% nervous system, 5% endocrine/ metabolic disorder and 5% had malignancy (Fig. 1). The prevalence of diagnosed chronic diseases by GPs was much higher than the prevalence of self-reported diseases (Fig. 1). The largest differences were observed between the prevalence of diagnosed and self-reported diseases in the mental (32%) and the endocrine and metabolic disorders (31%) (Fig. 1). Houseless people had the highest morbidity rates except for malignancies, and we found the largest proportion of individuals with multimorbidity among them (Table 2, Supplementary Table 1). Despite of the high prevalence of chronic diseases, homeless people were less likely to use medication (Table 2).

Homeless people visited a physician less frequently than the general population (Table 2). Ten percent of them had never visited a GP compared to 1% of the lowest income quintile of the general population and 0.6% of the general population. Although 70% of the participants had at least one chronic disease, only 41.4% of them visited a GP within a year. While 59% of the lowest income quintile and 50% of the general population had at least one chronic disease, almost all of them visited a physician in the previous 12 months (Table 2). Among those who had a chronic disease the highest proportion who never visited their GPs was in the group of roofless individuals (Table 2).

### Body mass index

The distribution of the body mass index was different in the three populations. Of the homeless participants, 35% were classified as overweight or obese, which was almost half of the prevalence in the two reference populations (Table 3). Although the prevalence of abnormally thin individuals (4%) was low in the homeless population, but it was still five times higher than in the general population (0.8%).

**Table 3:** Alcohol consumption, smoking habits and body mass index in the homeless population and in the general population in Hungary

	Homeless population						Lowest income quintile of the general population		General population	
	Roofless	Houseless	Insecure housing	Inadequate housing	P-value*	Overall		P-value†		P-value‡
**Alcohol consumption (%)**										
Abstinent	30.6	53.0	43.8	26.5	< 0.001	43.0	27.2	< 0.001	21.3	< 0.001
Occasional	25.0	19.8	18.8	28.6		22.1	35.7		41.8	
Moderate	4.0	6.5	2.1	12.2		6.0	10.2		13.9	
Heavy	40.4	20.7	35.5	32.7		28.9	26.9		23.1	
***Amount of wine per day (%)***										
Nothing	62.1	80.2	77.1	65.3	0.007	73.3	89.0	< 0.001	91.1	< 0.001
0.1–3 dl	4.0	5.2	6.3	2.0		4.6	5.1		4.2	
3–9 dl	11.3	5.6	4.2	12.2		7.7	4.2		4.0	
More than 10 dl	22.6	9.1	12.5	20.4		14.3	1.7		0.7	
***Amount of beer per day (%)***										
Nothing	59.7	77.2	79.2	67.3	< 0.001	71.5	86.4	< 0.001	89.5	< 0.001
0.1–3 dl	2.4	0.9	0	0		1.1	1.3		1.3	
3–9 dl	12.1	13.4	2.1	6.1		11.0	6.2		6.1	
More than 10 dl	25.8	8.6	18.8	26.5		16.3	6.2		3.1	
***Amount of spirits per day (%)***										
Nothing	64.5	81.5	83.3	69.4	0.03	75.7	91.2	< 0.001	94.8	< 0.001
Maximum 3 shots	23.4	13.8	10.4	18.4		16.6	7.4		4.8	
3–9 shots	5.6	1.3	4.2	6.1		3.3	0.7		0.2	
10 shots or more	6.5	3.4	2.1	6.1		4.4	0.6		0.1	
**Cigarette smoking (%)**										
Never	9.7	10.3	8.3	18.4	0.6	10.8	32.4	< 0.001	41.6	< 0.001
Former	8.1	6.9	10.4	4.1		7.3	21.8		25.7	
Current	82.3	82.8	81.3	77.6		81.9	45.8		32.7	
***Number of cigarettes (%)***										
0–5 cigarettes	18.6	15.5	15.4	5.3	0.002	15.3	7.8	0.002	9.2	0.001
6–10 cigarettes	19.6	21.1	12.8	50.0		22.8	20.7		23.5	
11–15 cigarettes	17.6	22.2	25.6	15.8		20.6	22.0		21.7	
16–20 cigarettes	21.6	24.7	30.8	15.8		23.6	32.6		31.2	
> 20 cigarettes	22.5	16.5	15.4	13.2		17.7	16.9		14.3	
**Body mass index (%)**										
Abnormally thin	2.6	4.4	6.4	4.4	0.6	4.1	1.8	< 0.001	0.8	< 0.001
Normal	65.2	56.8	59.6	69.6		60.9	36.3		31.1	
Overweight	22.6	22.8	21.3	15.2		21.7	35.5		41.0	
Obese	9.5	16.1	12.7	10.8		13.3	26.5		27.1	

*Comparing the different homeless categories

† Comparing the overall homeless category to the lowest income quintile of the general population

‡ Comparing the overall homeless category to the general population

Within the homeless population, the prevalence of BMI> 25 was the highest among people who lived in temporary accommodation (houseless).

### Alcohol consumption

Difference was found in the frequency of alcohol consumption in the homeless population and in the general population. Surprisingly, abstinence was reported most commonly (43%) in the homeless population. This rate was 21% in the general population and 27% in the lowest income quintile of the general population (Table 3). The homeless population was characterized by the lowest proportion of occasional and moderate drinkers, and the highest proportion of heavy drinkers. The prevalence of consumption of more than 1 l wine or more than 3 shots spirits daily was more than 25 times higher in the homeless population than in the general population, and almost six times higher than in the lowest income quintile of the general population. Of the heavy drinkers 38% was roofless people.

### Smoking habits

Smoking is universal in the homeless population, there was no difference between the homeless ETHOS categories, the majority of them were current smokers (82%). This rate was more than double than in the general population (33%), and also much higher than in the lowest income quintile of the general population (46%) (Table 3). The prevalence of heavy smokers (smoking more than 20 cigarettes per day) was also higher in the homeless population (18%) than in the general population (14%) and it was similar in the lowest income quintile of the general population (17%). The highest prevalence was detected among the roofless people (23%).

## Discussion

Our aim was to study the health problems and health behaviour of the Hungarian homeless people who use the homeless care system with the comparison of the health data of the general Hungarian population. Our results were presented in the context of ETHOS typology of homelessness to demonstrate the health-related heterogeneity between the subgroups of homeless population.

In Europe, except Finland and Norway, all countries have seen a rising trend in homelessness [31]. It is estimated that there are approximately 30,000 houseless and roofless people in Hungary. Additionally, there are approximately 300,000 households, around 8% of all Hungarian households, which can be categorized as insecure tenures and inadequate forms of housing [32]. It is well known that poor housing is associated with infectious and chronic diseases, injuries, poor nutrition, and mental disorders [33].

Overall, our study clearly shows that besides all other problems, homeless people have severe health problems compared even to the most disadvantaged segment of the general Hungarian population. Of them 70% suffer from chronic diseases, which is 1.5 times higher than in the general population. The prevalence of multimorbidity was almost three times higher than it was observed in the general population. In line with previous findings, their unhealthy lifestyle characterized by high proportion of heavy smoking and drinking further worsen the prognosis of their conditions [21, 24, 34]. Despite of the presence of the chronic disease they hardly consult a GP, therefore their diseases are not monitored or controlled. Seeking health care is usually not a priority for them as they struggle with other life threatening conditions such as lack of food and lack of shelters. Furthermore they often experience barriers in accessing health services [35]. The low frequency of GP visits and the high rate of untreated chronic diseases among the homeless people compared to the average population is particularly noteworthy in light of the fact that in Hungary in addition to the generally available health care, a special health service for homeless people exists within the social care, which includes “Homeless GP care”, “Recovering care”, “24-hour health centers” and “mobile medical services”. Homeless GP care is available in almost all county capitals in Hungary, usually in the building of the local homeless service providers, so the location of the GP office is in a familiar environment and it can be easily accessed by the homeless people. There might be many reasons besides access barriers why disadvantaged people use health services less than it would be expected from their health status. For example, a recent study by Marek et al. (2020) pointed out that among Romas „‘Seeking medical attention too late’ as well as ‘neglecting and leaving diseases untreated’ are healthcare-related behaviours”. Hesitancy to use health services might be due to fear of illness, pain, or death or previous perceived or experienced discrimination [36].

Homeless people generally have poor health literacy [37], and in many instances they are not even aware of the health problems they have, which may explain the observed differences between prevalence of GP diagnosed and the self-reported diseases. These differences were particularly visible in our study in the case of endocrine and metabolic diseases, diseases of the digestive system, and mental disorders. In contrast to the previous findings, the prevalence of the neoplasm was relatively low among them [4]. Several studies have been demonstrated that case management, health education, improvement of social skills, and social support could improve the health literacy, the health services utilization and the adherence to the therapy [38–42].

Poor health literacy, lack of preventive interventions, and health promotion programs together with the underutilization of health services have critical implications. There is a rapidly growing need for nursery type of services for homeless people. Ageing of the population puts further pressure for the service providers.

While the homeless people do not form one single population, most of the published results show a uniform and significant difference between the health problems of the homeless and the general population. ETHOS typology is particularly useful for addressing different social and health care needs within the homeless population which has important implications for the improvement of services and the mode of delivery for them.

Using the ETHOS definition, ‘roofless’ people include those “living without a fixed shelter and people in emergency accommodation provided only on a night-by-night basis” (FEANTSA, 2005) In general, poor health status and unhealthy behaviour and environment of roofless people is well documented, but the health of people in other categories of homelessness is much less studied. Although our research confirms that the prevalence of heavy drinking and smoking are the highest among roofless people, not all health problems were the most prevalent among them. According to the FEANTSA report, health problems and health care needs of roofless people may have been exaggerated in the cross-sectional studies because it overrepresents those homeless people who experience living rough for long-term with severe mental illness and drug and alcohol problems [1]. A larger self-reliant transitionally homeless group experiences living rough for shorter periods also exist with more favourable health indicators [1]. Our data on the presence of chronic diseases support this type of distribution in the roofless group, in addition with the concordance of a recent Spanish research, in which less than half of the examined population was heavy drinker [43].

Contrary to previous findings, houseless people, people in temporary accommodation had the highest rate of chronic diseases in our study. Furthermore, the highest prevalence of mental disorders was among the houseless group, of whom many were suffering from cardiovascular and gastrointestinal diseases, too. The health problems of ‘houseless’ populations are less studied. One reason for the highest occurrence of chronic diseases among them might be that they were on average older than the individuals in the other groups, therefore the age-related chronic diseases were overrepresented in this group. The sustained stays in temporary accommodation provide regular access to GP consultation, thus their diseases could have been diagnosed, which may also contribute to the observed high prevalence rates. According to the Act III of 1993 on social administration and social services and the Act XLII of 1999 on the protection of non-smokers and on certain rules of consuming and distributing tobacco products is forbidden to bring alcohol and intoxicants to the temporary accommodations, and to enter the accommodation in a state strongly influenced by alcohol or drugs. Smoking is only allowed outside of the buildings. Among them, the proportion of non-smokers and abstinent was the highest which might be due to the institutional policy, but the highest rate of obesity was detected in this group too.

Inadequate and insecure housing conditions (like mould growth, indoor air pollution, inefficiency of heating systems, or lack of sanitation amenities) may trigger many health problems [44]. It has already been reported that the prevalence of mental disorders, respiratory and gastrointestinal diseases are high in people living in insecure and inadequate living conditions [33, 45, 46]. The prevalence of chronic diseases was the lowest in these two groups in our study compared to the roofless and houseless groups.

Self-rated or self-reported health is good indicators of health status [47] but it has rarely been used in studies of health and homelessness. In our study, homeless people reported their health status poorer than the two reference populations. Similar results were found by Wagner et al. (2014) and Lebrun-Harris LA et al. (2013) [34, 48]. The prevalence of self-reported health status varied by homeless sub-population. Deterioration in self-reported health is associated with insecure housing [49]. We found a social gradient in self-reported health status from roofless people to individuals living in insecure living conditions.

In Hungary, the non-housing-focused, staircase-oriented support does not allow for differentiated care, only few innovative housing-led solutions are available. The accommodation-based services include emergency accommodation, temporary hostels, rehabilitation institutions, old people’s homes. Non-accommodation-based services are food service, soup kitchens, day centres, street outreach, emergency phone lines, healthcare centres. These services are run by NGOs, churches and municipalities. Municipalities with populations of above 30,000 are required to provide emergency shelters and temporary hostels. In municipalities with populations of between 10,000 and 30,000, food distribution and day-centre services are part of the legal duties of local government.

Although providing shelter and adequate housing are of great importance for homeless people, it often overshadows other issues including health-related problems, which are very relevant for them. In the last ten years a progress took place in homeless health research and homelessness related policymaking [50–53]. Since 2010 homelessness has become an important topic in the EU with the social inclusion target of the Europe 2020 Strategy to lift at least 20 million people out of the risk of poverty and social exclusion [50]. In concordance with the strategy the Joint Report on Social Protection and Social Inclusion (2010) and the Social Investment Package (2013) called on Member States to develop their comprehensive homelessness strategies [51, 52]. Furthermore, the European Pillar of Social Rights recognizes the right of the homeless to housing and assistance [53]. Despite the strategic approaches and integrated strategies for fighting homelessness and housing exclusion of the European Commission, unfortunately Hungary is still lacking a comprehensive and consistent strategy and policy framework to address homelessness. In line with the European strategy on homelessness and housing exclusion the FEANTSA conducting and disseminating research and data collection in the EU to promote a better understanding on homelessness. But there is still a need for addressing this issue and providing robust data about the adverse health circumstances of homeless people highlighting the different health and social needs of the subgroups among the homeless population so that the services could be tailored to their needs.

Our study has some limitations. We used convenient sampling in the Homeless Health Survey, thus our study is not representative of the total Hungarian homeless population. Nevertheless, the subgroup of homeless people who utilize any kind of homeless care services was well covered by our study. The vast majority of roofless and houseless people are known by the homeless care providers and therefore were well represented in our study. Regarding people belonging to inadequate or insecure housing categories, a much smaller proportion of them are known by these services. They constitute a larger group, many of them are hidden, and there is not any sampling frame to select an adequate representative sample of them. We included only those people in the study who contacted the homeless care services. This had an impact on the results that only the most disadvantaged subgroup of inadequate or insecure housing categories were represented in our study. As our study was cross-sectional, people being homeless for long – chronic homeless people – could have been over-represented in the study.

## Conclusions

Overall, this study describes the health problems and the health-related behaviour of the Hungarian homeless people compared to the general population, its most disadvantaged segment, and by the ETHOS categorization. The health status and health behaviour of homeless people in general is much worse than the most disadvantaged people in the general population. They utilize health services much less, which further worsen their prognosis. A clear social gradient exists among homeless people, their subgroups are characterized by different levels of health problems and health needs as well. People living in houseless conditions (temporary institutions or shelters) were characterized with the highest prevalence of chronic diseases and multimorbidity.

Our results draw attention to the profound need of homeless people for specific interventions to improve their health. A prerequisite for this is the realization of the issue by caregivers, which requires sensitization and training. Homelessness legislation needs to be reviewed and modified in Hungary in order to improve the homeless care with special needs. The intersectoral collaboration and coordination between the social, health and labour sectors should be improved at the level of the central and local governments in line with integrated policies and actions to decrease the health inequalities and to develop a coherent and effective health promotion program for the homeless people in Hungary. Once this is achieved combined social and health interventions tailored to the specific needs of the different subgroups of homeless people could be the next step forward.

## Supplementary Information

**Additional file 1: Table S1.** Prevalence (%) of chronic diseases diagnosed by general practitioners in the Hungarian homeless population by ETHOS categories.

## Data Availability

The datasets used and/or analysed during the current study are available from the corresponding author on reasonable request.

## Abbreviations

FEANTSA: European Federation of National Organisations Working with the Homeless
ETHOS: European Typology on Homelessness and Housing Exclusion
EHIS2014: European Health Interview Survey 2014
EU: European Union
GP: General Practitioner
BMI: Body Mass Index
