# Supplementary material for: Health status and health behaviour of the Hungarian homeless people
Source: Arch Public Health. 2021 Feb 2;79:15. doi: 10.1186/s13690-021-00534-2 (PMC7852122; doi:10.1186/s13690-021-00534-2)
Supplement: Supplementary file 1 — Additional file 1: Table S1. Prevalence (%) of chronic diseases diagnosed by general practitioners in the Hungarian homeless population by ETHOS categories. [file 13690_2021_534_MOESM1_ESM.docx]

Supplementary Table 1. Prevalence (%) of chronic diseases diagnosed by general practitioners in the Hungarian homeless population by ETHOS categories

|  | **Rooflessness** | **Houselessness** | **Insecure housing** | **Inadequate housing** | **P-value^*^** |
| --- | --- | --- | --- | --- | --- |
| Cardiovascular diseases | 43.5 | 56.0 | 31.3 | 32.7 | 0.001 |
| Diseases of the digestive system | 33.9 | 53.0 | 31.3 | 20.4 | <0.001 |
| Mental disorders | 31.5 | 51.3 | 31.3 | 28.6 | <0.001 |
| Diseases of the respiratory system | 18.5 | 32.8 | 27.1 | 16.3 | 0.01 |
| Diseases of the musculoskeletal system | 25.0 | 29.3 | 27.1 | 18.4 | 0.44 |
| Diseases of the nervous system | 18.5 | 32.3 | 20.8 | 8.2 | 0.001 |
| Endocrine and metabolic diseases | 30.6 | 43.5 | 16.7 | 30.6 | 0.001 |
| Malignancy | 4.0 | 7.3 | 8.3 | 2.0 | 0.33 |

^*^P-value calculated by Chi-square test
